# Reply: SABRE for airway quantification in idiopathic pulmonary fibrosis: clarifications, limitations, and next steps

**DOI:** 10.1183/13993003.01897-2025

**Published:** 2025-11-20

**Authors:** Yang Nan, Federico Felder, Simon Walsh, Guang Yang

**Affiliations:** 1 Bioengineering Department and Imperial-X, Imperial College London, London, UK; 2 Royal Brompton Hospital, London, UK; 3 National Heart and Lung Institute, Imperial College London, London, UK; 4 School of Biomedical Engineering and Imaging Sciences, King's College London, London, UK

## Abstract

We thank Z. Zhang and co-workers, and T. Zhang for their thoughtful and constructive comments on our recent study of SABRE (Smart Airway Biomarker Recognition Engine) for airway quantification in idiopathic pulmonary fibrosis (IPF) [1]. Below, we provide a point-by-point response to the key issues raised.

## Shareable abstract

**SABRE is a robust imaging biomarker for airway quantification in idiopathic pulmonary fibrosis, while its full clinical adoption depends on prospective validation, broader clinical endpoints and integration into decision-making frameworks** https://bit.ly/3VVacfK

*Reply to Z. Zhang and co-workers, and T. Zhang:*

We thank Z. Zhang and co-workers, and T. Zhang for their thoughtful and constructive comments on our recent study of SABRE (Smart Airway Biomarker Recognition Engine) for airway quantification in idiopathic pulmonary fibrosis (IPF) [1]. Below, we provide a point-by-point response to the key issues raised.

*Selection/survivor bias*: We acknowledge the concern that retrospective registries may preferentially capture patients who survive long enough to undergo high-resolution computed tomography and follow-up. In our study, however, the enrolled patients had a maximum follow-up of over 10 years, with a mean survival of 4 years. Importantly, 12-month mortality was 10% and 12-month progression occurred in 20% of patients, indicating that patients with rapid progression were not systematically excluded and that the cohort captured a broad range of disease trajectories.

*Mixed training data and potential confounding*: It is correct that the SABRE training set included heterogeneous lung disease cohorts. We wish to clarify that this stage was focused on airway modelling – segmentation and structural quantification – rather than directly learning prognostic signals. In this context, inclusion of diverse phenotypes (*e.g.* nodules, emphysema, cancer, ground-glass opacities) was deliberate to enhance the robustness and generalisability of airway segmentation. Moreover, the branch-splitting algorithm was rule-based, derived from anatomical and clinical knowledge, and not subject to data-driven biases. Prognostic associations were assessed only in fibrotic interstitial lung disease (including IPF) cohorts, ensuring that survival signals reflect disease-specific pathways rather than confounding effects from other lung diseases.

*Lack of independent prospective validation*: We agree that retrospective fitting cannot replace independent, protocolised prospective validation, and acknowledge this as a limitation. Both the Australian Idiopathic Pulmonary Fibrosis Registry (AIPFR) and Open Source Imaging Consortium (OSIC) datasets are large, multicentre, multi-source registries, which mitigate, but cannot entirely eliminate, the risk of inflated performance estimates. We are currently preparing prospective validation studies that will directly address this issue.

*Limited clinical endpoints*: We also acknowledge that retrospective datasets restrict the range of available endpoints. While we used 12-month progression (≥10% forced vital capacity decline or ≥15% diffusing capacity of the lung for carbon monoxide decline) as a widely accepted and practical composite measure, other clinically meaningful outcomes, such as hospitalisation, acute exacerbation, initiation of oxygen therapy and patient-reported outcomes (*e.g.* the King's Brief Interstitial Lung Disease (K-BILD) health status questionnaire) were not available. We agree this is a limitation and plan to incorporate these endpoints in future prospective studies.

*Potential confounders (emphysema, inspiratory effort, comorbidities, treatment variables)*: As noted by T. Zhang, concomitant emphysema (CPFE phenotype) can influence airway calibre measurements. In our cohort, CPFE prevalence was <10% (radiological review), and sensitivity analyses excluding advanced emphysema cases yielded similar hazard ratios for airway metrics. Crucially, our airway metrics were obtained by normalising airway volumes with total lung volume (AV/LV), obtained *via* whole lung segmentation, specifically to mitigate variability due to inspiratory effort. Scans with poor inspiratory quality were excluded during pre-processing. Regarding unmeasured comorbidities and treatment adherence, as raised by Z. Zhang and co-workers, some variables (*e.g.* antifibrotic therapy status) were included in exploratory models; nevertheless, residual confounding is possible and will be addressed with richer longitudinal data in prospective studies.

*Clinical applicability and decision-making*: Importantly, in our study, risk stratification was explored using tertiles and the median of the SABRE signal, both of which effectively discriminated survival risk. However, we did not propose a fixed universal cut-off in this initial analysis, as the clinical utility of SABRE extends beyond defining a universal cut-off. In practice, SABRE can be used as a continuous covariate to refine multivariable models, to stratify patients by quantiles for triage and trial enrichment, and to monitor longitudinal change (ΔSABRE) as an early signal of progression. We will pre-specify risk bins for prospective validation, but emphasise that decision-making can already benefit from SABRE without relying on a single threshold.

*Segmentation performance*: T. Zhang highlighted the challenge of airway segmentation in severely fibrotic lungs and requested information on failure rates. As we have previously reviewed, airway segmentation in pathological conditions presents unique computational challenges that require robust algorithmic approaches [2, 3]. Meanwhile, we have also proposed several highly robust and accurate algorithms for airway modelling [4, 5]. In our study, the detection rates of airway branches were reported in the supplementary material, demonstrating that SABRE achieved >94% completion with manual review. Moreover, the external validation performed on IPF cohorts directly tested SABRE's robustness in complex fibrotic morphologies, supporting its stability in real-world conditions. We agree that it is important to incorporate a hybrid quality-control pipeline to flag low confidence cases in clinical practice.

In summary, we thank both groups of authors for their insightful and constructive feedback, which has provided valuable perspectives on the current limitations and future directions for SABRE research. Many of the suggestions align with our ongoing and planned work, demonstrating the shared commitment to advancing precision medicine in IPF.

The concerns raised highlight several critical areas that warrant further investigation. First, the need for prospective validation represents the most pressing next step, as retrospective analyses, despite their multicentre nature, cannot fully substitute for protocolised prospective studies with predefined endpoints and risk stratification criteria. Second, the incorporation of a broader range of clinically meaningful endpoints – including hospitalisation rates, acute exacerbations, oxygen therapy initiation, and patient-reported outcomes – will provide a more comprehensive assessment of SABRE's clinical utility beyond traditional physiological measures.

The technical robustness of SABRE, as evidenced by >94% completion rates in airway segmentation even in severely fibrotic lungs, provides a solid foundation for clinical implementation. However, we acknowledge that hybrid quality-control pipelines and automated flagging of low confidence cases will be essential for real-world deployment. The normalisation approach (AV/LV) and exclusion of poor quality scans represent important methodological strengths that address many potential confounders, though residual confounding from unmeasured variables remains a consideration.

Looking forward, our research agenda encompasses several key priorities: 1) multicentre prospective validation studies with pre-specified risk bins and clinical decision points; 2) longitudinal assessment of ΔSABRE as a dynamic biomarker for disease progression monitoring; 3) integration with existing clinical prediction models to enhance overall prognostic accuracy; 4) evaluation of SABRE's utility in clinical trial enrichment and therapeutic response monitoring; and 5) development of automated quality assurance frameworks (*e.g. via* data harmonisation [6] and quality control models [7]) for clinical deployment.

We believe these future refinements, informed by the valuable feedback received, will further establish SABRE as a clinically useful prognostic biomarker that can meaningfully impact patient care and clinical decision-making in IPF. The convergence of advanced imaging analytics with robust clinical validation represents a promising pathway toward personalised medicine in interstitial lung disease, and we remain committed to addressing the methodological and clinical challenges identified to realise this potential.

## Shareable PDF

10.1183/13993003.01897-2025.Shareable1 This PDF extract can be shared freely online.
Shareable PDF

ERJ-01897-2025.Shareable
