# Supplementary material for: Reply: SABRE for airway quantification in idiopathic pulmonary fibrosis: clarifications, limitations, and next steps
Source: Eur Respir J. 2025 Nov 20;66(5):2501897. doi: 10.1183/13993003.01897-2025 (PMC12645090; doi:10.1183/13993003.01897-2025)
Supplement: Supplementary file 1 [file ERJ-01897-2025.Shareable.pdf]

SHAREABLE PDF

Reply: SABRE for airway quantification in idiopathic pulmonary fibrosis: clarifications, limitations, and next steps

Yang Nan [1,2], Federico Felder [2], Simon Walsh [2,3] and Guang Yang [1,2,4]

[1]Bioengineering Department and Imperial-X, Imperial College London, London, UK. [2]Royal Brompton Hospital, London, UK. [3]National Heart and Lung Institute, Imperial College London, London, UK. [4]School of Biomedical Engineering and Imaging Sciences, King's College London, London, UK.

Corresponding author: Yang Nan (y.nan20@imperial.ac.uk)

Shareable abstract (@ERSpublications)
SABRE is a robust imaging biomarker for airway quantification in idiopathic pulmonary fibrosis, while its full clinical adoption depends on prospective validation, broader clinical endpoints and integration into decision-making frameworks https://bit.ly/3VVacfK

Cite this article as: Nan Y, Felder F, Walsh S, *et al*. Reply: SABRE for airway quantification in idiopathic pulmonary fibrosis: clarifications, limitations, and next steps. *Eur Respir J* 2025; 66: 2501897 [DOI: 10.1183/13993003.01897-2025].

This PDF extract can be shared freely online.

Copyright ©The authors 2025.

This version is distributed under the terms of the Creative Commons Attribution Licence 4.0.

Received: 5 Sept 2025
Accepted: 1 Oct 2025

Reply to Z. Zhang and co-workers, and T. Zhang:

We thank Z. Zhang and co-workers, and T. Zhang for their thoughtful and constructive comments on our recent study of SABRE (Smart Airway Biomarker Recognition Engine) for airway quantification in idiopathic pulmonary fibrosis (IPF) [1]. Below, we provide a point-by-point response to the key issues raised.
